# Genome-Wide Identification and Interaction Analysis of Turbot Heat Shock Protein 40 and 70 Families Suggest the Mechanism of Chaperone Proteins Involved in Immune Response after Bacterial Infection

**DOI:** 10.3390/ijms25147963

**Published:** 2024-07-21

**Authors:** Yuanwei Geng, Yuxuan Gai, Yanping Zhang, Shengwei Zhao, Anlan Jiang, Xueqing Li, Kaiqing Deng, Fuxuan Zhang, Lingling Tan, Lin Song

**Affiliations:** 1 School of Life Science, Qingdao Agricultural University, Qingdao 266109, China; 15698148630@163.com (Y.G.); gyx0903@163.com (Y.G.);; 2 Key Laboratory of Applied Mycology, Qingdao Agricultural University, Qingdao 266109, China; 3 Qingdao International Center on Microbes Utilizing Biogas, Qingdao Agricultural University, Qingdao 266109, China; 4 College of Entrepreneurship and Innovation, Qingdao Agricultural University, Qingdao 266109, China

**Keywords:** turbot, heat shock protein, molecular docking, *Aeromonas salmonicida*

## Abstract

Hsp40–Hsp70 typically function in concert as molecular chaperones, and their roles in post-infection immune responses are increasingly recognized. However, in the economically important fish species *Scophthalmus maximus* (turbot), there is still a lack in the systematic identification, interaction models, and binding site analysis of these proteins. Herein, 62 Hsp40 genes and 16 Hsp70 genes were identified in the turbot at a genome-wide level and were unevenly distributed on 22 chromosomes through chromosomal distribution analysis. Phylogenetic and syntenic analysis provided strong evidence in supporting the orthologies and paralogies of these HSPs. Protein–protein interaction and expression analysis was conducted to predict the expression profile after challenging with *Aeromonas salmonicida*. *dnajb1b* and *hspa1a* were found to have a co-expression trend under infection stresses. Molecular docking was performed using Auto-Dock Tool and PyMOL for this pair of chaperone proteins. It was discovered that in addition to the interaction sites in the J domain, the carboxyl-terminal domain of Hsp40 also plays a crucial role in its interaction with Hsp70. This is important for the mechanistic understanding of the Hsp40–Hsp70 chaperone system, providing a theoretical basis for turbot disease resistance breeding, and effective value for the prevention of certain diseases in turbot.

## 1. Introduction

The turbot, *Scophthalmus maximus*, is a significant economic species in aquaculture [1,2]. Stressors during turbot cultivation can be categorized into abiotic factors, such as high temperature, salinity, and water quality, and biotic factors, including bacterial, viral, and parasitic diseases, with bacterial infections being particularly common. Recent years have seen an increase in outbreaks of infections caused by *Aeromonas salmonicida*, emerging as a major disease threat [3]. Heat shock proteins (HSPs) have long been considered molecular chaperones that assist in the proper folding of proteins under high temperature stress [4,5,6]; however, increasing research has highlighted the pivotal role of HSPs in post-infection immunity [7,8,9,10,11]. Hsp40 and Hsp70, as a pair of molecular chaperones, typically interact structurally to function effectively [12]. Nevertheless, in the immune response to infection in turbot, the focus has predominantly been on the function of Hsp70, neglecting the interaction between this chaperone pair. This study will systematically identify the Hsp40 and Hsp70 families in turbot and analyze their interaction following *A. salmonicida* infection. This study aims to fill the gap in our understanding of the Hsp40 and Hsp70 interaction, offering a deeper comprehension of their mechanistic roles in fighting infections and managing stress in aquaculture environments.

Members of the Hsp40 family are also known as J proteins (DnaJ) as they all contain a highly conserved J domain [13]. Hsp40 can be classified into three groups: group A possesses the J domain, the Gly/Phe-rich region, and the cysteine repeats; group B possess the J domain and the Gly/Phe-rich region, but lack the cysteine repeats; and group C only possesses a helical J domain [5,14,15]. The J domain is a key site for the interaction of Hsp40 and Hsp70, which contains four α-helical structures. The second helix is enriched with lysines, a region rich in positively charged amino acid residues that are necessary for the ATPase activity of Hsp70. There is a tripeptide between the second and third helix consisting of histidine, proline, and aspartic acid (HPD) that constitutes its core structure [16,17]. It was recently found that *E. coli* DnaJ (Hsp40) cannot activate ATP hydrolysis of DnaK (Hsp70) by the J domain alone and that the ATPase stimulation requires a G/F-rich region [18]. Faust et al. revealed the NMR structure of the JD-GF domain of Hsp40 and found that upon binding to Hsp70, the G/F-rich region could form a stabilized α-helical structure [19]. Hsp70 are highly conserved in amino acid sequences and structures, and the main structure can be divided into four regions from the N-terminal to the C-terminal: a 40 kDa N-terminal ATPase domain (NBD), an 18 kDa substrate binding domain (SBD), a 15 kDa conserved peptide-binding functional domain, and a 10 kDa variable region at the C-terminal end, including an EEVD motif [20,21]. 

Hsp40 typically binds to heat shock protein 70 to facilitate ATP hydrolysis in order to perform its molecular chaperone function and participates in the next cycle upon completion. In the Hsp40–Hsp70 chaperone cycle, Hsp40 carries the substrate and stimulates the ATP hydrolysis to ADP on Hsp70, causing a conformational change in Hsp70 that allows it to bind tightly with the substrate. Subsequently, the nucleotide exchange factor (NEF) in turn stimulates the release of ADP from Hsp70 and facilitates the binding of a new round of ATP to the ATP-binding site on Hsp70, triggering the release of the substrate [12]. In addition, these processes are dependent on the ATPase activity of Hsp70, and Hsp40 is in turn thought to regulate how tightly Hsp70 binds to Hsp40-carrying substrates by modulating Hsp70 enzyme activity [22,23,24,25,26,27]. However, the mechanism by which Hsp40 regulates the ATPase activity of Hsp70 remains unclear. Faust et al. reported that the J domain as well as the GF domain of Hsp40 have important roles in the binding process with Hsp70 and that the binding of Hsp40 to Hsp70 requires the support of other components in addition to the roles of the J domain and the GF domain [19,28]. Therefore, we speculate that the binding site of Hsp40 to Hsp70 is not only present on the J domain. The specific binding sites of Hsp40 and Hsp70 as well as the sequence characteristics need to be further explored.

Since turbot is extremely demanding in terms of temperature and susceptible to a wide range of environmental stresses, the important functions of the HSPs’ family under different stresses make the regulation of their genes more pronounced in turbot, which can be investigated as a molecular biomarker of stress in fish. In addition, given the important functions of Hsp70 genes in response to environmental stresses and the abundant genome sequence resources, more and more Hsp70 gene families have been identified at the genome-wide level in a wide range of scleractinian fishes, and in recent years, the potential roles of these genes in responding to abiotic and biotic stresses have also been described [29,30,31,32]. Moreover, the high-quality genome [33] assemblies and multiple published RNA-seq dataset [34,35] resources about turbot in recent years have made it possible to systematically conduct identification, phylogenetic analyses, and functional research of Hsp40 and Hsp70 genes in turbot, which facilitates subsequent research.

In this study, genome-wide identification of the Hsp40 and Hsp70 families of heat shock proteins was performed, and the protein interaction between one pair of chaperones Dnajb1b and Hspa1a, which is involved in the immune response after bacterial infection, was analyzed.

## 2. Results

### 2.1. Identification of Hsp40 and Hsp70 Genes in the Turbot Genome

**Figure 1:**
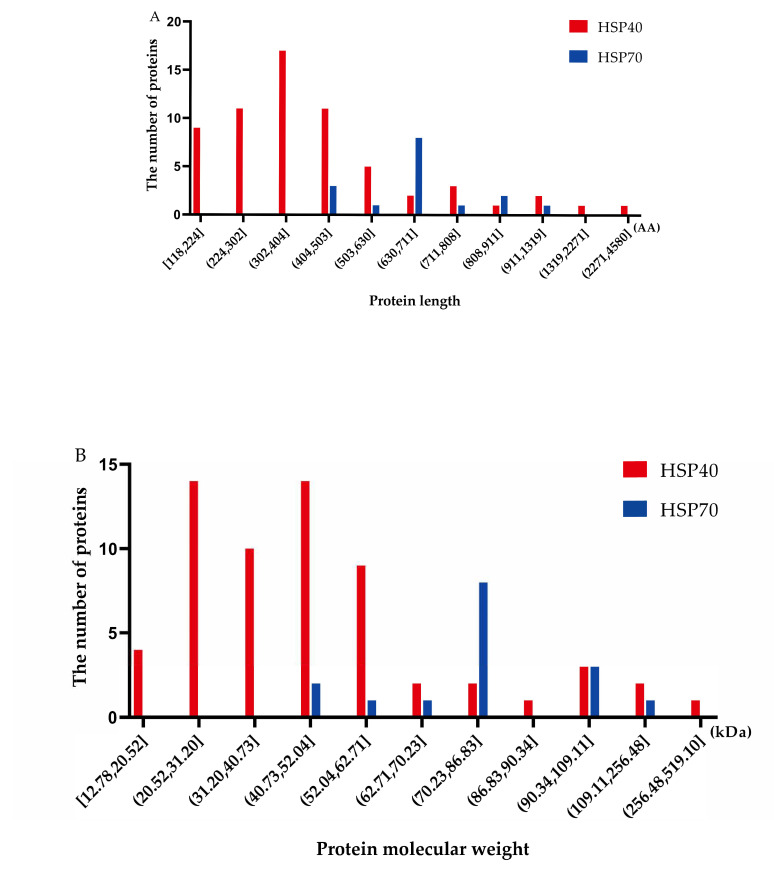

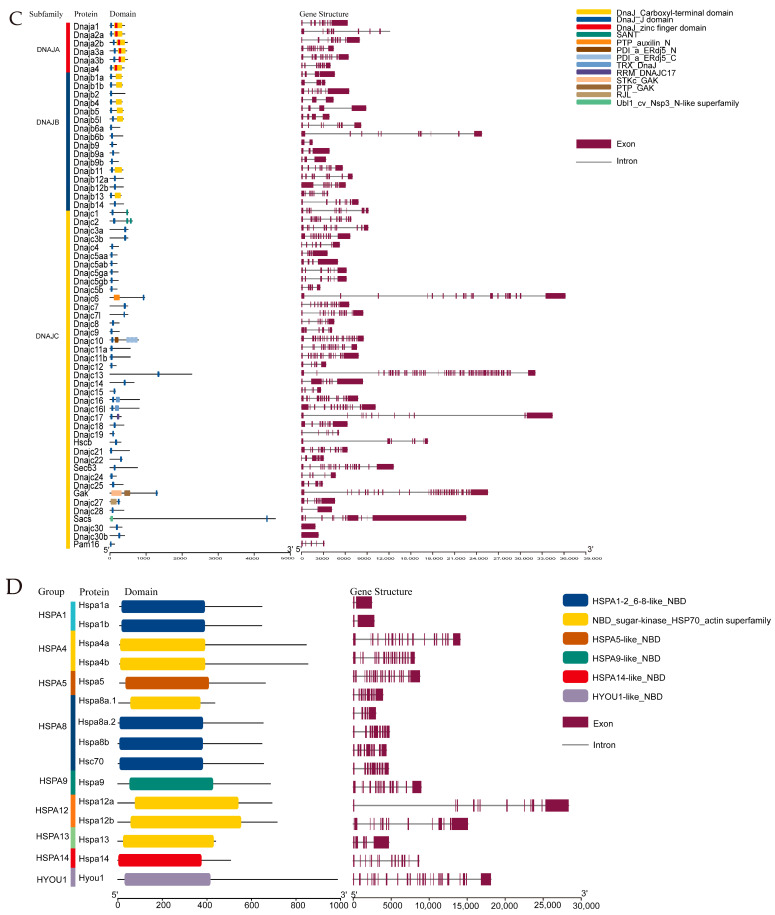
The characteristics and domains of Hsp40 and Hsp70 members in *S. maximus*. (**A**) Protein sequence length distribution; AA, amino acid; (**B**) the protein molecular weight distribution; (**C**) distribution of conserved domains and gene structure on the Hsp40; (**D**) distribution of conserved domains and gene structure on the Hsp70.

A total of 62 Hsp40 genes and 16 Hsp70 genes were found in the turbot genome based on the HMM search and domain analysis. As shown in Figure 1, the Hsp40 protein sequences vary from 118 AA to 4580 AA in length, and nearly 27.4% of the members were between 302 AA and 404 AA in length (Figure 1A). The protein molecular weights range from 12.78 kDa to 519.1 kDa and are mainly concentrated between 40.73 kD to 52.04 kDa (Figure 1B). The lengths of the Hsp70 protein sequence range from 439 AA to 986 AA, and the protein molecular weights extend from 47.64 kDa to 110.91 kDa, with a major concentration between 70.23 kDa and 79.47 kDa.

The distribution of conserved domains on the Hsp40 and Hsp70 proteins is illustrated in Figure 1C,D. The members of Hsp40 were classified into three subfamilies based on the Conserved Domains Database (CDD): DNAJA, DNAJB, and DNAJC. The DNAJA subfamily has J domain (JD), zinc finger domain, and carboxyl-terminal domain (CTD). The DNAJB subfamily has J domains, while not all members of DNAJB possess CTD. The DNAJC subfamily has a more diverse range of conserved domains; however, they all have J domains in common. In the Hsp70 protein family, several structurally similar and functionally related nucleotide binding domains (NBDs) are observed in each protein (Figure 1C,D). As shown in Figure 1C,D, the number of exons in Hsp40 and Hsp70 genes varied greatly from 1 to 57 and from 2 to 25. However, paralogous genes derived from the same group were shown to possess similar gene structures. For instance, both Dnajb5 and Dnajb5l contained three introns and four exons; both hspa1a and hspa1b in group HSPA1 contained only one intron and two exons, which was a composition obviously different from the members in the other groups (Table S1).

### 2.2. Chromosome Distribution Analysis

**Figure 2:**
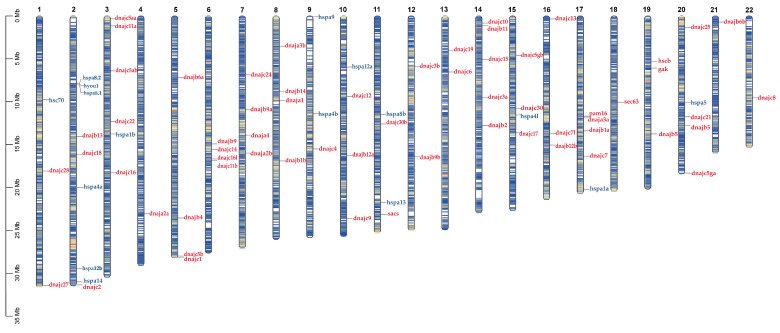
The genome positions of Hsp40 and Hsp70 members in *S. maximus*. The Hsp40 genes and the Hsp70 genes are labeled in red and blue respectively.

The 62 and 16 members of the Hsp40 and Hsp70 gene families are distributed on 22 chromosomes of the turbot reference genome (Figure 2). The results showed an average of three Hsp40 genes per chromosome. Among these chromosomes, the highest number of Hsp40 genes (five) was found on chromosomes Chr3 and Chr14, whereas the lowest number was found on chromosome Chr4, with only one Hsp40 gene. Hsp70 gene distribution also showed clustering on some chromosomes, such as six Hsp70 genes: hspa8a.1, hspa8a.2, hspa4a, hspa12b, hspa14, and hyou1, located on Chr2. In addition, two Hsp70 genes were observed on Chr9 and Chr11, respectively. The remaining six Hsp70 genes were distributed on different chromosomes, such as Chr1, Chr3, Chr10, Chr15, Chr17, and Chr20. 

### 2.3. Motif Analysis of the Identified Hsp40 and Hsp70 Proteins

To identify the conserved motifs within the turbot Hsp40 and Hsp70 families, MEME analysis was conducted. Five conserved motifs (named Motif1 to Motif5) were identified in each of the A, B, and C subfamily proteins of the Hsp40 family, and ten motifs (named Motif1 to Motif10) were identified in the Hsp70 family.

**Figure 3:**
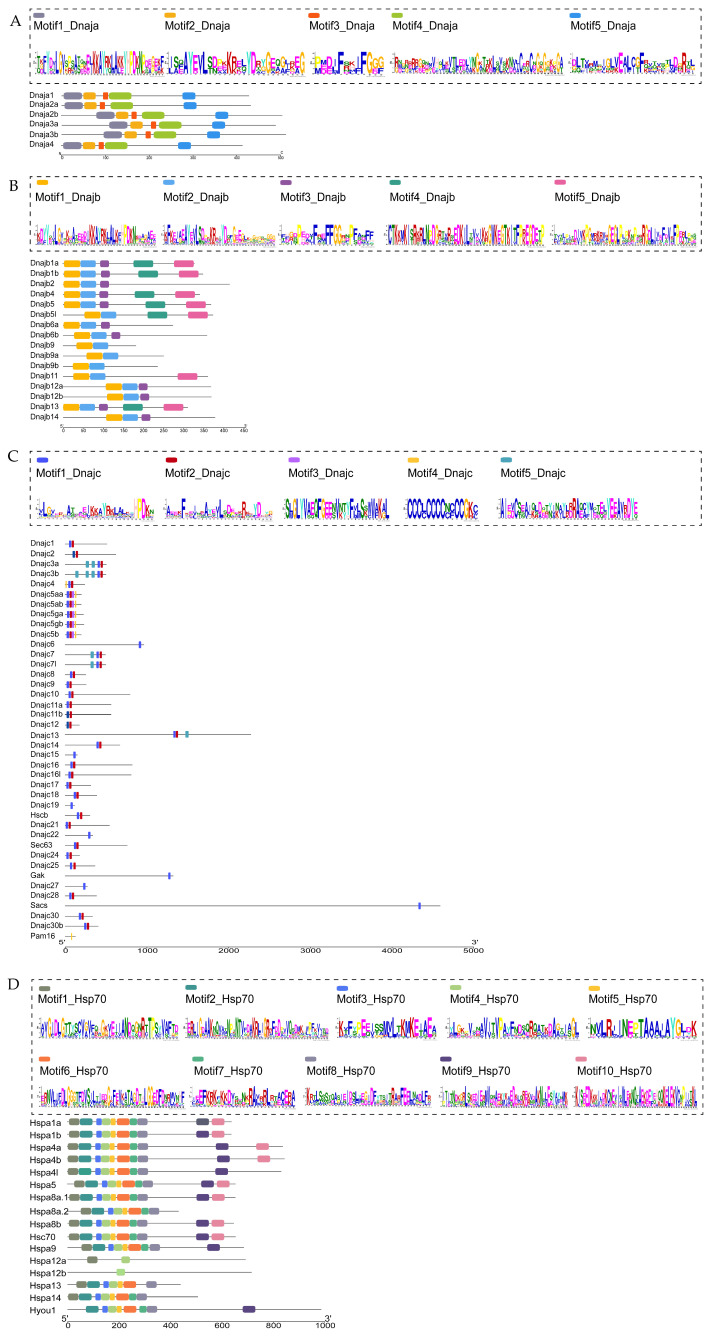
Conserved motif information for Hsp40 and Hsp70 proteins in *S. maximus*, and each colored rectangular box represents a motif. (**A**) Motif information in turbot Hsp40 family subfamily A; (**B**) motif information in turbot Hsp40 family subfamily B; (**C**) motif information in turbot Hsp40 family subfamily C; (**D**) motif information in turbot Hsp70 family.

The detailed information of Hsp40 and Hsp70 family motifs were listed. The results showed that the sequence lengths of the five motifs in subfamily A ranged from a minimum of 12 amino acids (Motif3) to a maximum of 50 amino acids (Motif4) (Figure 3A). The sequence lengths of the five motifs in subfamily B ranged from a minimum of 23 amino acids (Motif3) to a maximum of 50 amino acids (Motif4, 5) (Figure 3B). The sequence lengths of the five motifs in subfamily C ranged from a minimum of 15 amino acids (Motif4) to a maximum of 40 amino acids (Motif5) (Figure 3C). By comparing the three Hsp40 subfamilies, we found similar Motif1s containing the conserved tripeptide HPD in all three subfamilies, which is coincident with the conserved polypeptide sequence between the second and third alpha helices of the J domain. In addition, the motif corresponding to the GF-rich region was found in both subfamilies A and B (Motif3). No such motif was found in subfamily C. In Hsp70, only Motif4 was found in all the members, suggesting that Motif4 is a key sequence in the Hsp70 family (Figure 3D). This variable motif distribution may provide potent support for the phylogenetic classification and contribute to the functional diversity of the Hsp40 and Hsp70 protein families in turbot.

### 2.4. Phylogenetic Analysis

**Figure 4:**
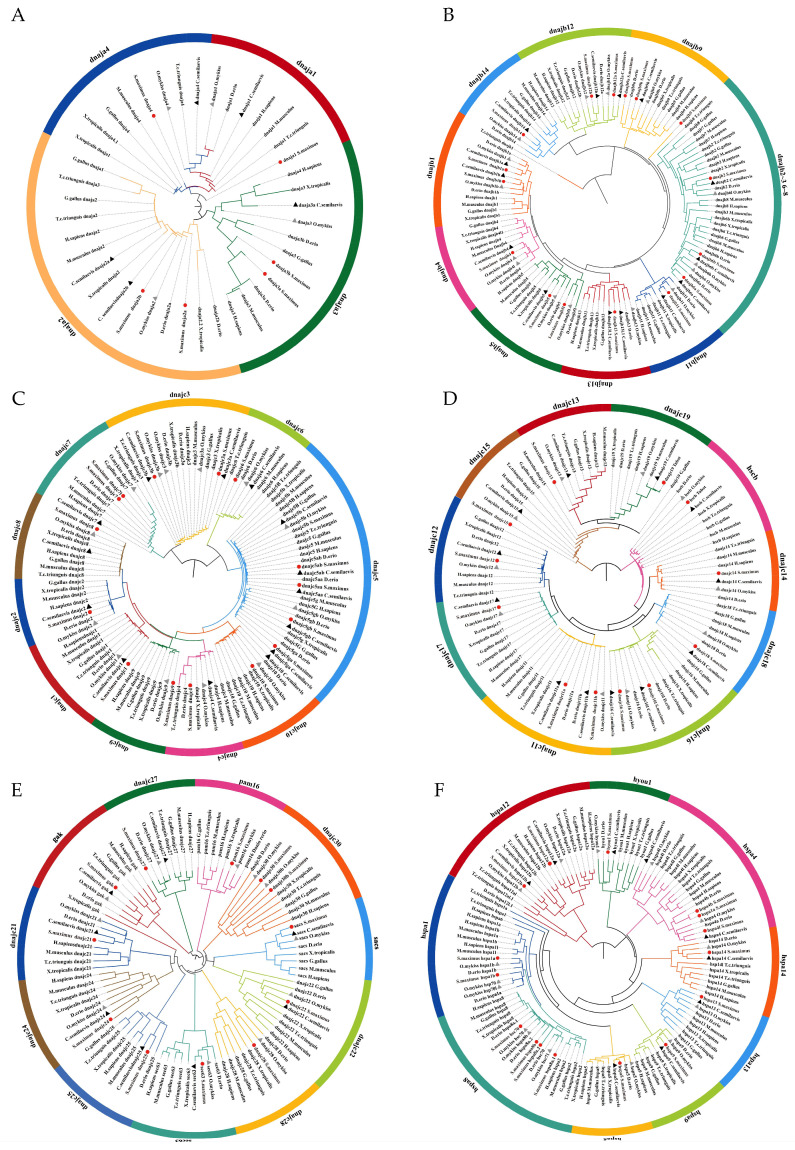
The phylogenetic tree constructed from Hsp40 and Hsp70 proteins in *S. maximus*, *C. semilaevis*, *O. mykiss*, *D. rerio*, *X. tropicalis*, *T. c. triunguis*, *G. gallus*, *M. musculus*, and *H. sapiens*. Different colors indicate different groups. The genes for turbot, half-smooth tongue sole, and rainbow trout are labeled with red dots and black and gray triangles, respectively. (**A**) Phylogenetic tree of subfamily A of the Hsp40 family; (**B**) phylogenetic tree of subfamily B of the Hsp40 family; (**C**) phylogenetic tree of dnajc1~dnajc10 in subfamily C of the Hsp40 family; (**D**) phylogenetic tree of dnajc11~dnajc20 in subfamily C of the Hsp40 family; (**E**) phylogenetic tree of dnajc21~dnajc30 in subfamily C of the Hsp40 family; (**F**) phylogenetic tree of Hsp70 family.

To understand the structural classification of the Hsp40 and Hsp70 gene families and clarify the phylogenetic relationships of Hsp40 and Hsp70 gene families among different vertebrate species, neighbor-joining (NJ) trees were constructed. Accession ID of the protein used in the tree is listed in Table S2. There are three subfamilies in the Hsp40 family, subfamily A, subfamily B, and subfamily C. Due to the large number of members in subfamily C, subfamily C was further divided into three groups (dnajc1–10, dnajc11–20, dnajc21–30) according to the order of sequence number for the phylogenetic tree construction. Hsp70 proteins of the selected vertebrates were constructed to build the phylogenetic tree. The results showed that subfamily A clustered into four clusters; subfamily B clustered into nine clusters; and subfamily C clustered into 31 clusters in the Hsp40 family (Figure 4A–E). The Hsp70 family clustered into nine clusters (Figure 4F). The results showed that turbot was more closely related to half-smooth tongue sole and rainbow trout.

### 2.5. Synteny and Gene Duplication Analysis

**Figure 5:**
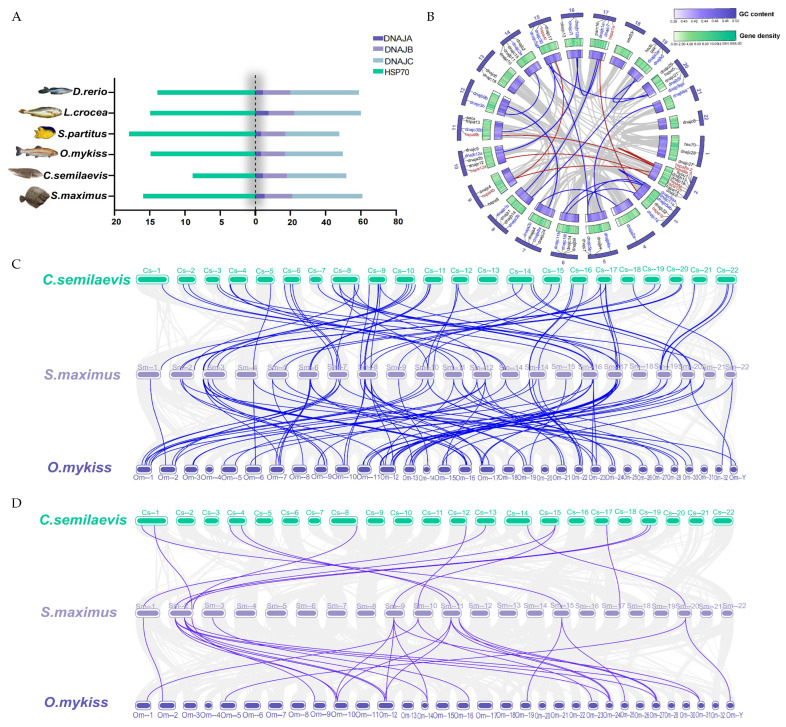
Duplication event analysis for the Hsp40 and Hsp70 gene families in the turbot genome and the synteny analysis between turbot and other fishes. (**A**) Number of Hsp40 and Hsp70 genes in six fish species; (**B**) the paralogues of Hsp40 and Hsp70 in the turbot genome—the blue and red lines indicate the Hsp40 and Hsp70 families members, respectively; (**C**) sytenic analysis of Hsp40 genes between *S. maximus*, *C. semilaevis* and *O. mykiss*—the blue lines indicate the Hsp40 family members in different species; (**D**) sytenic analysis of Hsp70 genes between *S. maximus*, *C. semilaevis*, and *O. mykiss*—the violet lines indicate the Hsp70 family members in different species.

We counted the number of Hsp40 and Hsp70 genes in zebrafish, large yellow croaker, damselfish, rainbow trout, half-smooth tongue sole, and turbot and found that damselfish had the highest number of Hsp70 genes, which was likely due to the fact that they live in the tropics. The number of Hsp70 genes in turbot is similar to that of rainbow trout and large yellow croaker, but it differs in the number of half-smooth tongue sole, which is also a Pleuronectiformes. From the evolutionary perspective, it could be due to the fact that turbot have acquired more anti-stress genes. As shown in Figure 5B, 13 paralogues of Hsp40 genes were found in the turbot genome, indicating duplication events might be involved. Moreover, four duplication events involving the Hsp70 gene was found. Among these duplication events, two pairs of duplicated genes in Hsp40 occurred on adjacent chromosomes, for example, dnajc7l on chromosome 16 and dnajc7 on chromosome 17.

To further understand the possible evolutionary events involving the Hsp40 and Hsp70 gene families in different teleost species, three comparative syntenic maps of turbot with half-smooth tongue sole and rainbow trout were constructed. The results showed that 59 Hsp40 orthologues and 13 Hsp70 orthologues were found between turbot and half-smooth tongue sole as well as 127 Hsp40 orthologues and 27 Hsp70 orthologues between turbot and rainbow trout. The number of homologous gene pairs between turbot and rainbow trout was significantly more than half-smooth tongue sole, probably because both turbot and rainbow trout are more favored to survive in colder temperatures.

### 2.6. Protein-Protein Interaction Analysis

**Figure 6:**
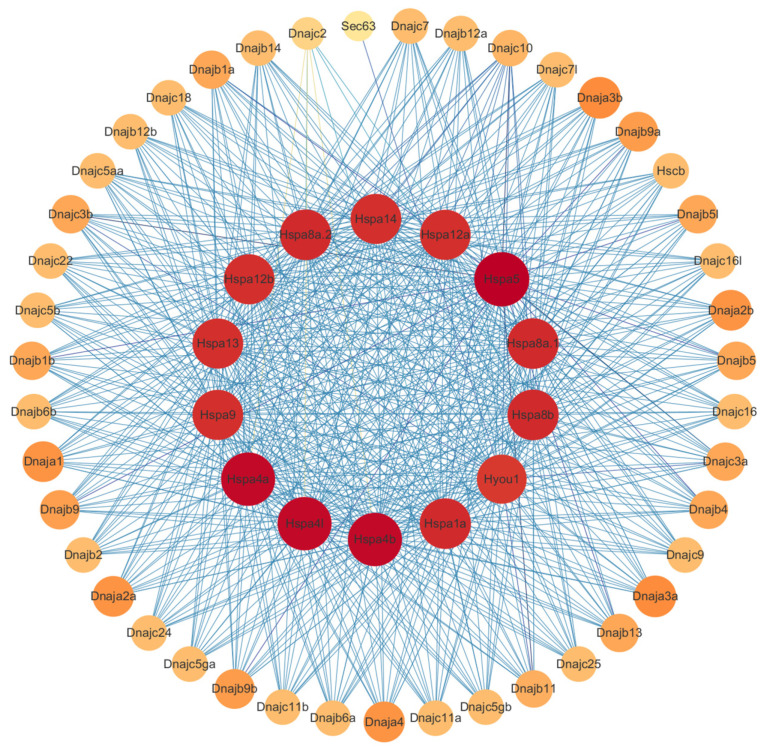
PPI network of the Hsp40 and Hsp70 proteins.

The 62 Hsp40 and 16 Hsp70 proteins were imported into the STRING database to obtain protein interaction network and screen for proteins with co-expression relationships. The analysis results were analyzed by Cytoscape 3.7.2, as shown in Figure 6 and Table S3 (Supplemental Information); nodes represent proteins, and edges represent relationships between proteins. The colors of nodes from yellow to red and the colors of edges from light blue to dark blue represent small to large degree values, respectively. The degree values of Hsp70 members were higher than those of Hsp40 by showing more edges and darker node colors in Hsp70 members.

### 2.7. Expression Analysis

**Figure 7:**
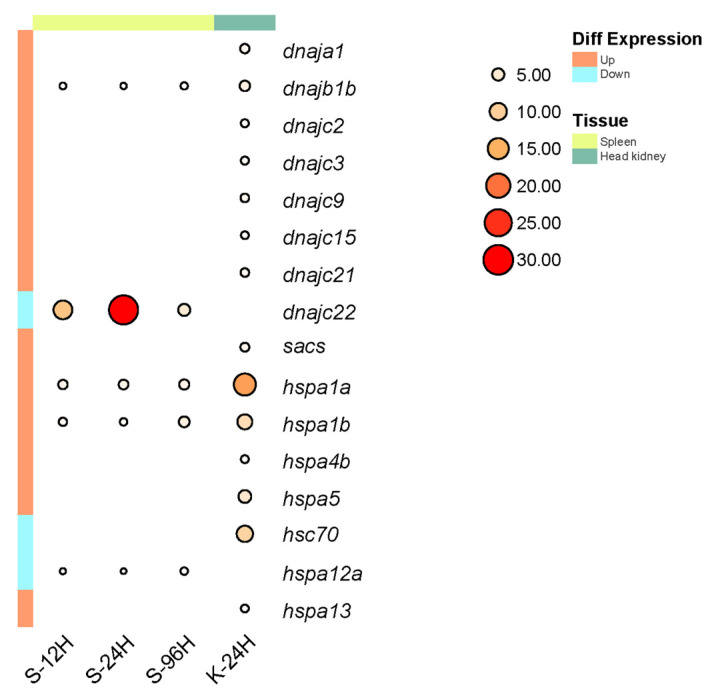
Expression analysis of differentially expressed genes of Hsp40 and Hsp70 families after infection with *A. salmonicida* in turbot.

To study Hsp40 and Hsp70 gene expression in turbot after infection with *A. salmonicida*, we analyzed their transcript abundance changes in transcriptome and RT–PCR data (Figure 7). After infection with *A. salmonicida*, five differentially expressed Hsp40 and Hsp70 genes were found in turbot spleen tissue, and fourteen differentially expressed Hsp40 and Hsp70 genes were discovered in turbot head kidney tissues. In the head kidney, only the expression of *hsc70* was down-regulated, while the expression of the other 13 genes was up-regulated. In the spleen, *dnajb1b*, *hspa1a*, and *hspa1b* showed an overall trend of up-regulation of expression, and the overall expression of *dnajc22* and *hspa12a* was down-regulated. In addition, *dnajb1b* and *hspa1a* show a more consistent expression trend compared to *hspa1b*, as their expression levels both increase with infection time. Therefore, we consider them co-expressed genes and hypothesize that they have interacting sites in the context of stress response between Hsp40 and Hsp70.

### 2.8. Molecular Docking Analysis

**Figure 8:**
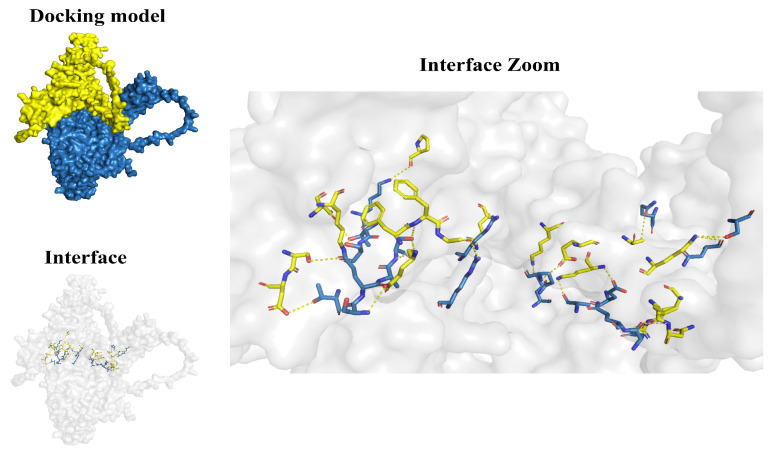
Dnajb1b and Hspa1a protein molecular docking model. Surface diagram of the docking model and their interfacing residues between Dnajb1b and Hspa1a protein (Dnajb1b, yellow; Hspa1a, blue; hydrogen bond interaction, dotted line).

To investigate the protein interaction sites of the co-expressed genes *dnajb1b* and *hspa1a*, we conducted molecular docking analysis. Hydrogen bonds in the secondary structure of protein are important for maintaining structural stability. The shorter the bond distance, the greater the bond energy. We found the amino acid residue sites and hydrogen bond distances where Hsp40 and Hsp70 interact with each other by PDBePISA, and screened the sites that can form relatively stable interactions according to the hydrogen bond distances. As shown in Figure 8 and Table S4 (Supplemental Information), molecular chaperones Dnajb1b and Hspa1a formed a few groups of hydrogen bonds through amino acid residue sites, such as LYS 21-THR 297 (distance = 2.25 Å) and GLU 105-ALA 282 (distance = 2.51 Å), revealing that proteins Dnajb1b and Hspa1a formed a stable protein docking model.

**Figure 9:**
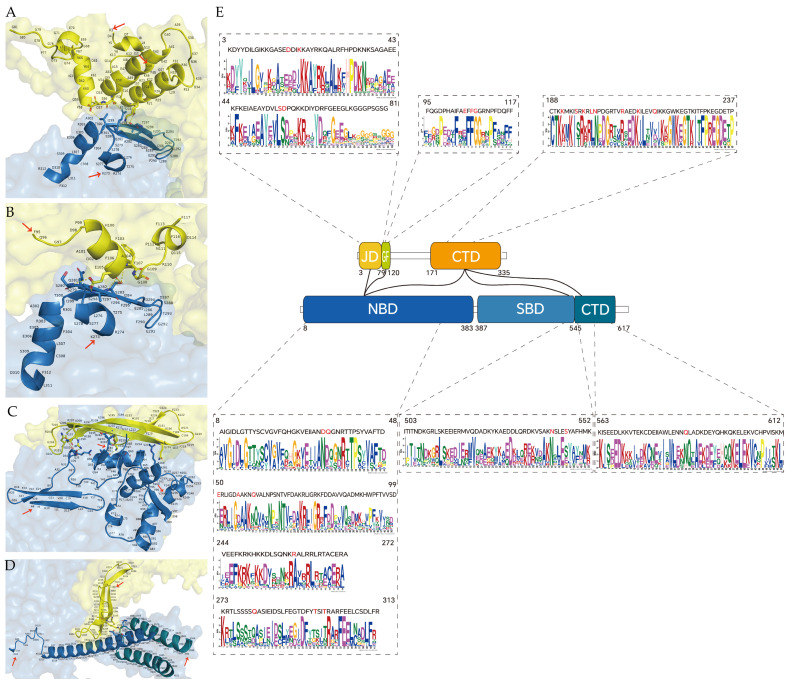
Dnajb1b and Hspa1a protein molecular docking model combined with motif analysis (Dnajb1b, yellow; Hspa1a, blue; hydrogen bonding interactions, dashed lines). (**A**) Molecular structure of the JD of Dnajb1b interacting with the NBD of Hspa1a; (**B**) molecular structure of the GF-rich regions of Dnajb1b interacting with the NBD of Hspa1a; (**C**) molecular structure of the CTD of Dnajb1b interacting with the NBD of Hspa1a; (**D**) molecular structure of the CTD of Dnajb1b interacting with both the SBD and the CTD of Hspa1a; (**E**) domain interactions between Dnajb1b and Hspa1a and the motif where the interactions amino acid sites are located.

When combining domain and motif analysis (Figure 9), certain amino acids in the JD and GF-rich regions of Dnajb1b can bind to specific amino acid sites within the same motif on the NBD of Hspa1a. D18, K21, S56, and D57 are binding sites in Hsp40 for Hsp70NBD. Among them, D18 and K21 are within a motif containing the conserved tripeptide HPD, and these two amino acids both bind to T297 of the NBD of Hspa1a. E105 and F107 are amino acids in the GF-rich region of Dnajb1b that bind to the NBD of Hspa1a. E105 can bind to three amino acids in the NBD, namely S280, A282, and S283, while F107 can only bind to S283. The CTD domain of Dnajb1b contains amino acids that bind to all three domains of Hspa1a. K191, L199, N200, R207, K211, Q216, and E232 are binding sites in the CTD of Dnajb1b for the NBD of Hspa1a. K197 is a binding site in the CTD of Dnajb1b for the SBD of Hspa1a, and S195 is a binding site in the CTD of Hsp40 for the CTD of Hspa1a. 

## 3. Discussion

Hsp40 and Hsp70, as a pair of molecular chaperones, play a crucial role in regulating cellular protein homeostasis and participating in the immune activities of organisms [36,37]. However, in turbot, systematic analysis of Hsp40 and Hsp70 is limited, and their interaction sites remain unclear. In our study, we identified 62 Hsp40 genes and 16 Hsp70 genes. Based on the conserved domain database (CDD), we classified Hsp40 into three subfamilies: A, B, and C, and Hsp70 into nine groups. Additionally, we constructed six phylogenetic trees and identified five conserved motifs in the three subfamilies of Hsp40 and ten conserved motifs in Hsp70. In the syntenic analysis, we studied the synteny of genes within turbot and the synteny between turbot and other species, such as half-smooth tongue sole and rainbow trout. In the expression analysis, we constructed a protein–protein interaction (PPI) network for Hsp40 and Hsp70, identifying differentially expressed Hsp40 and Hsp70 genes in the spleen and head kidney of turbot following infection by *Aeromonas salmonicida*. We selected a pair of Hsp40 and Hsp70 proteins that exhibited interaction and co-expression for molecular docking analysis to further explore their role in the immune response. This study focuses on addressing the gap in the prediction of interaction sites between Hsp40 and Hsp70 in turbot, potentially aiding in the development of new immunoregulatory strategies or treatment. These findings provide essential foundational information for research on the structure and function of heat shock proteins and may guide related genetic engineering and drug development projects.

Based on the constructed Hsp40 and Hsp70 phylogenetic trees, it is evident that fish and terrestrial vertebrates are evolutionarily related to each other. However, due to their distinct living environments, the affinities between fish and terrestrial vertebrates differ significantly, with fish species being more closely related to each other. The phylogenetic tree reveals a close relationship between turbot and zebrafish, rainbow trout, and half-smooth tongue sole. This, combined with the synteny analysis, further confirms the close affinity between turbot and these species. Notably, the number of orthologous gene pairs between turbot and rainbow trout was significantly higher than that of half-smooth tongue sole and zebrafish for the Hsp40 and Hsp70 families. Therefore, when researching relevant vaccine adjuvants for turbot, we might consider referring to the research findings related to half-smooth tongue sole to provide new insights for the development of vac-cines against bacterial diseases in turbot.

In addition to homologues, the number of genes varies from species to species. Differences in the number of genes may be the result of gene duplication or gene loss during evolution. Gene duplication is the primary mechanism for gene family expansion and provides the raw material for the evolution of new gene functions. For example, in the constructed developmental-evolutionary tree, it can be found that *dnajb1* has only one in humans, mice, chickens, three-toed turtles, and tropical clawed frogs but contains two genes (*dnajb1a*, *dnajb1b*) in zebrafish, rainbow trout, and half-smooth tongue sole, which are fishes. It was further confirmed that *dnajb1a* and *dnajb1b* are a set of duplicate genes in the synteny analysis. In addition, a significant up-regulation of *dnajb1b* expression was found in turbot after infection with *A. salmonicida*, and it was hypothesized that the duplication of *dnajb1b* was for better adaptation to environmental stresses in the aquatic environment.

In expression analysis, it can be found that *dnajb1b*, *hspa1a*, and *dnaja1b* show very similar changes in expression over time after infection with *A. salmonicida* and they all have a tendency toward up-regulated expression. In addition, upon combining the protein interaction analysis graphs (Figure 6) of Hsp40 and Hsp70, it can be found there is a strong interaction between *dnajb1b* and *hspa1a*. Therefore, based on the co-expression trend between them, *dnajb1b* and *hspa1a* are presumed to be one pair of chaperone molecules. 

Based on previous studies on the interaction between Hsp40 and Hsp70, researchers usually focus only on the interaction between the JD domain of Hsp40 and Hsp70 [38,39,40,41,42,43,44,45,46,47,48]. However, through molecular docking analysis, we have also discovered that the CTD of Dnajb1b can bind to Hspa1a and even interact with three domains of Hspa1a. Surprisingly, most of the binding sites between these domains are located within the conserved motifs we have identified. These conserved motifs may provide potential information for identifying molecular targets of interaction. Additionally, we identified a highly conserved peptide motif in the CTD domain of Dnajb1b, where certain amino acids can bind to amino acids in all three domains of Hspa1a (Figure 9). A study has shown that the subfamily B of Hsp40 has a self-inhibition mechanism different from subfamily A [14]. Under normal conditions, the JD of Hsp40 is in a self-inhibited state and does not bind to Hsp70. When the CTD of Hsp40 binds to the CTD of Hsp70, the JD is released from self-inhibition and binds to Hsp70. In our analysis, there are 22 pairs of interacting amino acids between Dnajb1b and Hspa1a (Table S2), with half of them being pairs of amino acids in the CTD of Dnajb1b interacting with amino acids in Hspa1a. This highlights the significant role of the CTD of Hsp40. Moreover, according to Table S2, the hydrogen bond lengths between certain amino acids in the CTD of Dnajb1b and amino acids in the NBD of Hspa1a (e.g., LYS 211-ALA 56, distance = 2.38 Å; LYS 211-GLU 50, distance = 2.49 Å) and those between amino acids in the JD of Dnajb1b and amino acids in the NBD of Hspa1a (e.g., LYS 21-THR 297, Distance = 2.25 Å) are relatively short, forming stable binding models. This further demonstrates that the CTD of Hsp40 plays a crucial role in its interaction with Hsp70, and certain conserved amino acid motifs in the CTD may function similarly to the HPD conserved tripeptide in the JD.

Hsp40 and Hsp70 are widely present and highly conserved protein families in evolution from bacteria to mammals. Recently, Hsp40 and Hsp70 have been increasingly used in the treatment and prevention of fish diseases [49,50,51,52,53,54,55] and even in the human diseases, such as cancer [56,57,58,59], neurodegeneration [60,61,62], and autoimmune diseases [63,64,65,66,67]. Most of these diseases are caused by the inefficiency of HSPs to correct the misfolded proteins. The conserved motifs on Hsp40 interacting with Hsp70 identified in this paper may provide some molecular targets for improving the efficiency of Hsp40–Hsp70 interaction and the theory basis for disease treatment.

## 4. Materials and Methods

### 4.1. Identification and Characterization of Hsp40 and Hsp70 Genes in Turbot

The turbot reference genome sequence, annotation files, and protein sequences were collected from NCBI (https://www.ncbi.nlm.nih.gov, accessed on 1 March 2023). The turbot Hsp40 and Hsp70 protein sequences were used as a query database, and the turbot amino acid sequences were searched using the BLASTp program with default parameters. Moreover, the candidate protein sequences were submitted to the Conserved Domains Database (CDD) to further confirm the existence of conserved domains with an e-value of 10^−4^. The exon–intron structure of turbot Hsp40 and Hsp70 genes was visualized using the Gene Structure Display Server (GSDS, version 2.0, http://gsds.cbi.pku.edu.cn, accessed on 6 August 2022) with the genome GTF file.

### 4.2. Chromosomal Distribution Analysis of Hsp40 and Hsp70 Genes

The turbot genome file and genome Generic Feature Format (GFF) file were collected from NCBI. Information on chromosome length and gene position was obtained from the genome Generic Feature Format (GFF) file, and then the genes were visualized using the gene location visualize function in TBtools (v1.098761) [68]. 

### 4.3. Motif Analysis of Hsp40 and Hsp70

Motif analysis of Hsp40 member classifications was performed using the MEME program (http://meme-suite.org/tools/meme, accessed on 30 March 2023). Conserved motifs of Hsp40 and Hsp70, with the maximum number of motifs, was set to five and ten, respectively. 

### 4.4. Phylogenetic Analysis of Hsp40 and Hsp70 Proteins

Hsp40 and Hsp70 proteins from the turbot and other selected vertebrate species were aligned using ClustalW [69] and MUSCLE [70]. Then, we constructed the neighbor-joining (NJ) phylogenetic trees with the pairwise deletion option based on the Jones–Taylor–Thornton (JTT) amino acid substitution model, using MEGA11.0 software [71] with bootstrap test of 1000 replicates. EvolView (v2.0) (https://www.evolgenius.info/evolview, accessed on 28 December 2023) was used to visualize the phylogenetic tree. 

### 4.5. Synteny Analysis of Hsp40 and Hsp70 Genes

Gene duplication analysis of *Hsp40* and *Hsp70* genes was performed using TBtools software to identify duplication events among these *Hsp40* and *Hsp70* genes using the TBtools with the Graphics–Comparative Genomics–One Step MCScanX function [68].

Using the MCScanX program of Tbtools, we identified regions associated with Hsp40 and Hsp70 members with synteny among zebrafish, rainbow trout, and half-smooth tongue sole, and the related figures were generated by using TBtools software (Version 1.098761) [68,72]

### 4.6. Interaction Analysis of Hsp40 and Hsp70

Interaction analysis of Hsp40 and Hsp70 proteins was performed by String (http://cn.string-db.org, accessed on 22 April 2023). The protein interaction relationships were retrieved. The combined score from the export file was imported into Cytoscape to build a PPI network diagram [73].

### 4.7. Expression Analysis of Hsp40 and Hsp70

Two sets of transcriptomic data for *Hsp40* and *Hsp70* genes after infection with *Aeromonas salmonicida* were collected from the researches of Ting Xue et al. [34] and A. Romero et al. [37]. The 0 h (control) samples and the experiment groups of 12 h, 24 h, and 96 h samples35ere collected from the researches of Ting Xue et al. [34]. The 0 h (control) samples and the experiment group of 12 h samples were collected from the researches of A. Romero et al. [35].

The FPKM value was used to estimate the gene expression level, and the differentially expressed genes were obtained by DESeq with *p*-value < 0.05 and |log2FC| ≥ 1.

### 4.8. Molecular Docking of Dnajb1b-Hspa1a

Molecular Docking Analysis: Rigid protein–protein docking was performed between Dnajb1b and Hspa1a to investigate the relationships by using GRAMM-X (http://gramm.compbio.ku.edu/, accessed on 20 October 2023). The protein domains of Dnajb1b and Hspa1a were obtained from the Protein Data Bank PDB database (http://www.rcsb.org/, accessed on 20 October 2023), which was sourced from the Alpha fold [74]. PyMOL (Version 2.4), and PDBePISA (https://www.ebi.ac.uk/pdbe/pisa/, accessed on 20 October 2023) was used to investigate protein–protein interactions and further visual analysis [75]. Hydrogen bonds between residues with a bond distance of less than 5 Å are selected as stable interaction pairs.

## 5. Conclusions

We have identified 62 *Hsp40* genes and 16 *Hsp70* genes in the turbot genome. Their regulation pattern was related to tissue and time. Most of the *Hsp40* and *Hsp70* genes were up-regulated, and only a few of them were down-regulated when infected with *A. salmonicida*. According to the expression pattern, we built a relatively stable molecular model between Hsp40 and Hsp70, which revealed their interaction sites among each domain. The carboxyl-terminal domain of Hsp40 plays a crucial role in its interaction with Hsp70, and some conserved amino acid motifs in the carboxyl-terminal domain may be functionally similar to the conserved tripeptide of HPD in the J domain. This is important for the mechanistic understanding of the Hsp40–Hsp70 chaperone system and the development of small molecule inhibitors targeting Hsp70 or Hsp40, providing a theoretical basis for turbot disease resistance breeding and effective value for the prevention of certain diseases in turbot.

## Data Availability

The original contributions presented in the study are included in the article/Supplementary Materials, further inquiries can be directed to the corresponding author.

## Supplementary Materials

The following supporting information can be downloaded at https://www.mdpi.com/article/10.3390/ijms25147963/s1.
